# Facile Preparation of High Strength Silica Aerogel Composites via a Water Solvent System and Ambient Pressure Drying without Surface Modification or Solvent Replacement

**DOI:** 10.3390/ma14143983

**Published:** 2021-07-16

**Authors:** Dongxuan Du, Fengqi Liu, Yonggang Jiang, Junzong Feng, Liangjun Li, Jian Feng

**Affiliations:** Science and Technology on Advanced Ceramic Fibers and Composites Laboratory, National University of Defense Technology, Changsha 410073, China; ddx369@126.com (D.D.); liufengqi13@nudt.edu.cn (F.L.); junzongfeng@nudt.edu.cn (J.F.); liliangjun101@gmail.com (L.L.)

**Keywords:** silica aerogel, composite, thermal conductivity, ambient pressure drying, high strength

## Abstract

To further reduce the manufacturing cost and improve safety, silica aerogel composites (SAC) with low density and low thermal conductivity synthesized via ambient pressure drying (APD) technology have gradually become one of the most focused research areas. As a solvent, ethanol is flammable and needs to be replaced by other low surface tension solvents, which is dangerous and time-consuming. Therefore, the key steps of solvent replacement and surface modification in the APD process need to be simplified. Here, we demonstrate a facile strategy for preparing high strength mullite fiber reinforced SAC, which is synthesized by APD using water as a solvent, rather than using surface modification or solvent replacement. The effects of the fiber density on the physical properties, mechanical properties, and thermal conductivities of SAC are discussed in detail. The results show that when the fiber density of SAC is 0.24 g/cm^3^, the thermal conductivity at 1100 °C is 0.127 W/m·K, and the compressive strength at 10% strain is 1.348 MPa. Because of the simple synthesis process and excellent thermal-mechanical performance, the SAC is expected to be used as an efficient and economical insulation material.

## 1. Introduction

Silica aerogel is a new type of nanomaterial with a 3D nanoporous network structure. It was originally developed by Kistler in 1931 [1], and has attracted widespread attention due to its low density, low thermal conductivity, and high specific surface area [2,3,4]. The preparation process of aerogels usually involves sol–gel, aging, and supercritical drying [5,6]. The supercritical drying process usually adopted requires a high temperature and high pressure (the supercritical point of ethanol is 240 °C, 6.3 MPa, and the supercritical point of carbon dioxide is 31 °C, 7.38 MPa), which is costly, dangerous, and restricts the large-scale continuous industrial production of aerogels. Therefore, ambient pressure drying (APD) has received great attention [7]. However, early APD includes solvent replacement and surface modification, and the waste liquid produced in this process is difficult to be recycled and utilized, resulting in great waste and environmental pollution [8,9,10]. Since then, researchers have prepared silica aerogels using methyltrimethoxysilane (MTMS) as a precursor by APD without solvent replacement [11,12,13,14,15,16], but aerogels have poor mechanical properties and are prone to brittle fracture during use [17,18,19]. Our research group [20] used MTMS as the precursor and mullite fiber as the reinforcing phase to prepare an aerogel insulation composite material in the early stage, which has a low thermal conductivity (0.0403 W/m·K at room temperature and 0.101 W/m·K at 1100 °C), but its strength is not strong enough to resist external vibration and compression during use (0.108 MPa at 10% strain). In addition, flammable and explosive ethanol is used in the preparation process, which is dangerous and not friendly to the environment.

In this paper, the high strength aerogel insulation composites were prepared with MTMS as the precursor and mullite fiber as the reinforcing phase by a APD process; no ethanol, no solvent replacement, and no surface modification were involved. The effects of fiber density on the physical properties, mechanical properties, and thermal conductivities of silica aerogel insulation composites were investigated. This work will provide an important basis for the economical, efficient, and green preparation of high-performance thermal insulation materials.

## 2. Materials and Methods

MTMS, urea, and cetyltrimethylammonium bromide (CTAB) were all purchased from Shanghai Maclin Biochemical Technology Co., Ltd. (Shanghai, China). Acetic acid was purchased from Sinopharm Holding Chemical Reagents Co. Ltd. (Shanghai, China). Mullite fiber parts provided by Shandong Luyang Co., Ltd. (Zibo, China). None of the reagents were further purified.

MTMS was added into a 0.01 M acetic acid aqueous solution. Then, CTAB and urea were added and strongly stirred for 4 h to enhance the hydrolysis of MTMS and to obtain the silica sol. The molar ratio between MTMS, H_2_O CTAB, and urea is 1:8:0.05:0.7. Next, the prepared mullite fiber parts (different fiber densities at 0.20, 0.24, 0.26, 0.30, and 0.32 g/cm^3^) were impregnated with the above sol under vacuum conditions. The whole system was sealed tightly in 60 °C water to form a gel. Upon gelation, a small amount of deionized water was added to protect the gel. After aging in a water bath at 60 °C for 48 h to promote the cross-linking and strengthening of the nano skeleton, it was dried at ambient pressure for 8 h at 60 °C, 80 °C, 100 °C, 110 °C, and 120 °C, separately, to obtain the aerogel composite. The gradual drying step was done to ensure the integrity of the nano structure of the aerogel matrix. Then, the prepared samples were heat treated in a muffle furnace at 700 °C for 2 h (the rate of heat treatment is 5 °C/min) to obtain the final SAC.

The volumetric density of the silica aerogel composites was measured with the Archimedes method. The morphology was observed by scanning electron microscopy (TESCAN MIRA3 Brno, Czech Republic). A universal testing machine (WDW Model 100, Jinan, China) was used to test the compressive strength. The thermal conductivity of SAC at room temperature and high temperatures were measured with a heat flow meter (ASTM-E1530, New Castle, DE, USA) and hot plate meter (YB/T4130-2005, Luoyang, China), respectively. Mercury intrusion porosimeter (Autopore IV 9510, Norcross, GA, USA) was used to determine the pore size distribution. Nitrogen sorption analysis (Quantachrome autosorb-IQ2-MP, Boynton Beach, FL, USA) was used to characterize the BET (Brunner–Emmet–Teller) surface area and nano pore size of the SACs.

## 3. Results and Discussion

**Figure 1:**
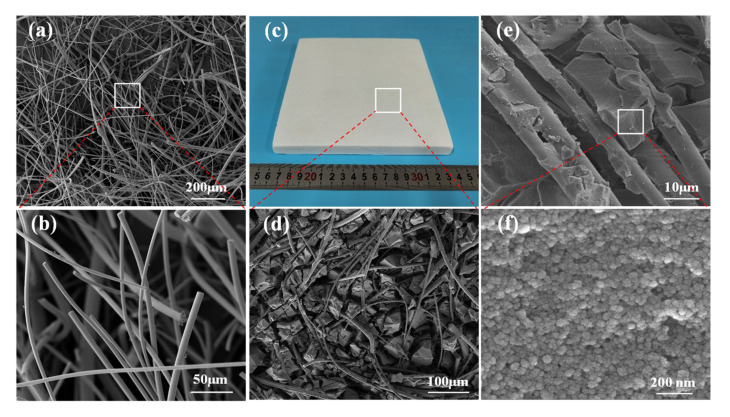
(**a**,**b**) SEM images of pure mullite fiber, (**c**) photographs of SAC, and (**d**–**f**) SEM images of the SAC.

The microscopic morphology of the mullite fiber is illustrated in Figure 1a,b, as lots of fibers go through each other and wind around each other. From the SEM, the average fiber length is 1.5 mm and the average fiber diameter is 5 μm. As can be seen from Figure 1c, the silica aerogel composite is well formed with smooth surface and no obvious crack. The SEM pictures (Figure 1d,e) show that the aerogel as the matrix fills the whole space in blocks, and the fiber runs through the aerogel matrix as the reinforcing phase. Moreover, the fiber and aerogel were closely bonded and had a good compatibility. Figure 1f reveals that the aerogel matrix is composed of many nanoparticles packed together, and many nanoscale gaps are formed between them.

**Figure 2:**
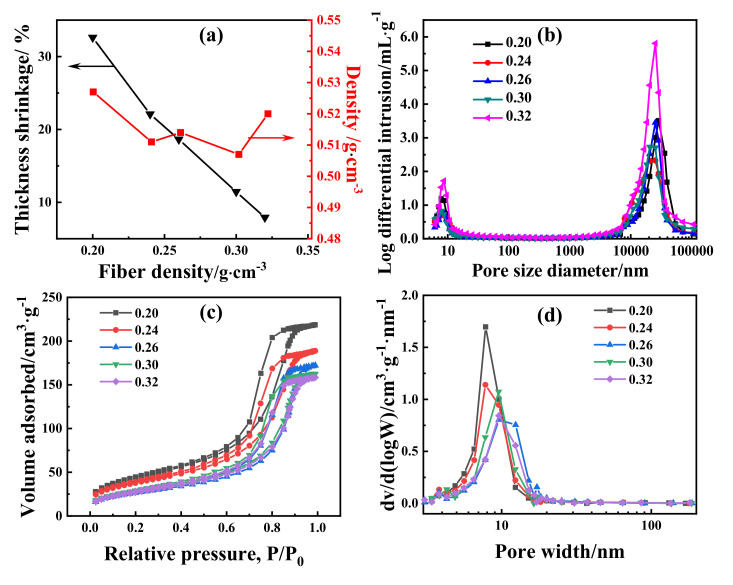
(**a**) Density and thickness shrinkage, (**b**) MIP pore size distribution, (**c**) NS isotherms, and (**d**) NS pore size distribution of SAC.

There was no visible shrinkage in the plane direction of the composite, and the shrinkage in the thickness direction is shown in Figure 2a. With the increase in fiber density, the shrinkage of the silica aerogel composite decreased continuously, from 32.61% at 0.20 g/cm^3^ to 7.92% at 0.32 g/cm^3^. Because of the interaction between the shrinkage of the aerogel and the fiber expansion, the density of the mullite fiber reinforced aerogel composites did not change significantly and fluctuated in the range of 0.51~0.53 g/cm^3^.

In order to analyze the pore size distribution of the composite material, mercury intrusion porosimeter (MIP) and nitrogen sorption (NS) methods were employed. Figure 2b illustrates the MIP pore size distribution curves of the composites with different fiber densities. It can be found that the pore size distribution curves of the composites have two peaks, indicating that the materials have two pore size structures: micron pore and nano pore, and the diameters of the nano pore and micron pore are concentrated on about 10 nm and 20 μm, respectively. The NS isotherms and the corresponding pore size distribution of the SACs are shown in Figure 2c,d. All of the SACs display type IV isotherms with a hysteresis loop according to the IUPAC (International Union of Pure and Applied Chemistry) classification, which indicates that there are nanopores between 2–50 nm in the SACs [21]. The NS pore size distribution revealed that the nanoscale pore structure around 10 nm of SACs was consistent with the SEM and MIP analyses. The detectable ranges of NS and MIP were 4–3 × 10^5^ nm and 0.35–100 nm, respectively, and the results from NS were more reliable than MIP within the pore width of 0.35–100 nm in this case [22,23,24,25]. So, we adopted the result from NS for the range of nano pores and MIP for the micron pores. Furthermore, the results from MIP in the range of nano pores testify to the results from the NS on the other side. All of the nano pores in the SACs were from the aerogel matrix. With the increase in the fiber density, the proportion of aerogel decreased, and as a result, the number of nano pores in the composites decreased, and the sample with density of 0.32 g/cm^3^ had the least number of nano pores. The BET specific surface areas of the SACs were 158.758, 137.108, 96.962, 104.433, and 99.849 m^2^/g for fiber density at 0.20, 0.24, 0.26, 0.30, and 0.32 g/cm^3^, respectively. Micron pores in the SACs were from two parts: gaps between fibers and gaps between fiber and aerogel. The increase in the fiber density led to the elevation of gaps between fibers. However, the variation of gaps between the fiber and aerogel was hard to analyse. What is more, the density of SACs also affected the micron pores, a high density usually resulted in more micron pores in this case. As a result of all of the above factors, with the increase in fiber density, the micron pores showed the same trend with densities of SACs, decreased first and then increased, and the sample with a density of 0.24 g/cm^3^ had the least number of micron pores.

**Figure 3:**
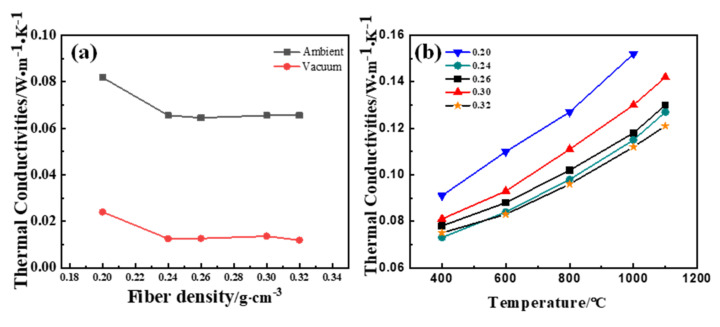
The thermal conductivity of SAC at (**a**) room temperature and (**b**) high temperatures.

The influence of the fiber density on the thermal conductivity of SAC was conducted and is shown in Figure 3. It be seen from Figure 3a that the vacuum thermal conductivity and ambient thermal conductivity of the aerogel decreased at first and then remained unchanged with the increase of fiber density. The thermal conductivity of SAC at an atmospheric pressure reaches the lowest value of 0.05806 W/m·K at 0.24 g/cm^3^, and the difference between the two curves is considered to be the thermal conductivity contributed by the gaseous phase, including the gaseous thermal conductivity at room temperature and the effect of gas–solid coupling [26].

Figure 3b depicts that the thermal conductivity of the silica aerogel composite continuously increased with the rise of temperature [18,27,28]. This phenomenon is attributed to the rapid increase in radiant thermal conductivity at high temperatures. With the increase of fiber density, the thermal conductivity of the silica aerogel insulation composites increased firstly, then decreased, and then increased. At 400 °C, the sample with a density of 0.24 g/cm^3^ showed the lowest thermal conductivity (0.073 W/m·K), and it had the best thermal insulation performance (0.121 W/m·K) when the density was 0.32 g/cm^3^ at 1000 °C.

**Figure 4:**
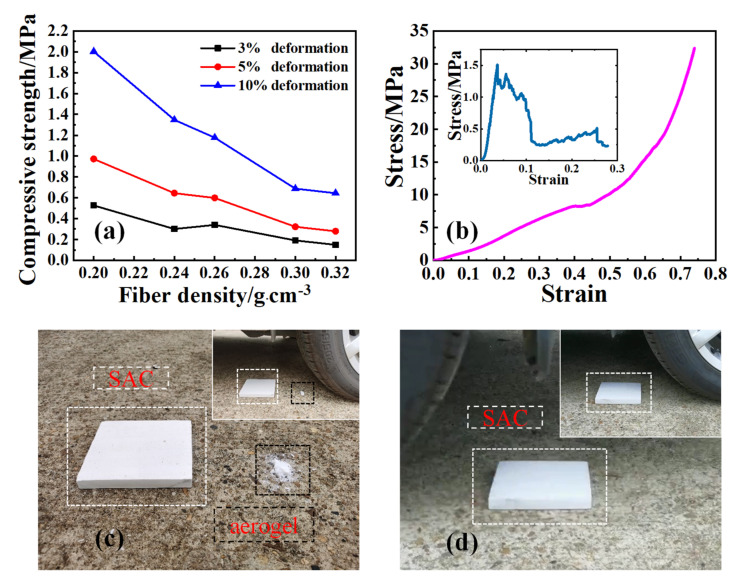
(**a**) Compressive strength as functions of the fiber density of SAC, (**b**) compressive stress–strain curve of SAC and aerogel (inset), (**c**) photos of the first car pressure test, and (**d**) photos of the second car pressure test.

As can be seen from Figure 4a, with the increase in fiber density, the compressive strength of the composite material decreased significantly. The compressive strength of the 10% strain decreased from 2.003 MPa (fiber density is 0.20 g/cm^3^) to 0.644 MPa (fiber density is 0.32 g/cm^3^). The reason is that under the same volume condition, with the increase of fiber density, the proportion of high-strength aerogel matrix in the composite material decreased, which made it unable to provide a strong support for the composite material. The comprehensive performance of SAC was best at a fiber density of 0.24 g/cm^3^, and its compressive strength at 10% strain was 1.348 MPa, which was 1146% higher than that of the same type of aerogel insulation composite [20]. Strong aerogels and toughened fibers play an important role in the preparation of regular-shaped and high-strength aerogel composites.

As can be seen from the compressive stress–strain curves of pure aerogel (inset diagram in Figure 4b) and the SAC (Figure 4b), the fracture of the pure aerogel before strain at 5% means terrible brittleness, but for SACs, it can still withstand stress without fracture after strain at 70%, which means good toughness rather than brittleness. In order to show the good toughness of the SAC more visually, we made “car pressure tests” (let a car run over the samples) on the SAC and a piece of corresponding aerogel. Figure 4c,d show the photos after the first/second car pressure test, while the insets in which show the photos before the tests. As in Figure 4c, for the first test, after the impact from the tyre of the car, the aerogel collapsed while the SAC remained intact. In the second test, the SAC after the first test was turned over and went on the second test. After the second test, it still remained intact without fracture, which means the SAC showed a certain degree of toughness on the whole.

## 4. Conclusions

In this paper, the mullite fiber reinforced silica aerogel insulation material was synthesized by the method of APD with water as the only solvent, without surface modification or solvent replacement. The effects of the fiber density on the physical properties, mechanical properties, and thermal conductivity of SAC were studied. With the increase in fiber density, the shrinkage and the compressive strength of SAC decreased obviously. In addition, the increase in fiber density caused the porosity and thermal conductivity to decrease first and then increase. In summary, when the fiber density was 0.24 g/cm^3^, the sample possessed a low high-temperature thermal conductivity (0.127 W/m·K at 1100 °C) and excellent compressive strength (1.348 MPa at 10% strain). These desirable features confirm the suitability of SAC aerogels prepared by APD technology as a high-performance and economical thermal insulation material.

## Data Availability

Data sharing is not applicable.
